# Data Mining for Identifying Novel Associations and Temporal Relationships with Charcot Foot

**DOI:** 10.1155/2014/214353

**Published:** 2014-04-27

**Authors:** Michael E. Munson, James S. Wrobel, Crystal M. Holmes, David A. Hanauer

**Affiliations:** ^1^Division of Metabolism, Endocrinology and Diabetes, Department of Internal Medicine, University of Michigan Medical School, 24 Frank Lloyd Wright Drive, Lobby C, Suite 1300, Ann Arbor, MI 48106, USA; ^2^Department of Pediatrics, University of Michigan Medical School, 5312 CC, SPC 5940, 1500 E. Medical Center Drive, Ann Arbor, MI 48109, USA

## Abstract

*Introduction*. Charcot foot is a rare and devastating complication of diabetes. While some risk factors are known, debate continues regarding etiology. Elucidating other associated disorders and their temporal occurrence could lead to a better understanding of its pathogenesis. We applied a large data mining approach to Charcot foot for elucidating novel associations. *Methods*. We conducted an association analysis using ICD-9 diagnosis codes for every patient in our health system (*n* = 1.6 million with 41.2 million time-stamped ICD-9 codes). For the current analysis, we focused on the 388 patients with Charcot foot (ICD-9 713.5). *Results*. We found 710 associations, 676 (95.2%) of which had a *P* value for the association less than 1.0 × 10^−5^ and 603 (84.9%) of which had an odds ratio > 5.0. There were 111 (15.6%) associations with a significant temporal relationship (*P* < 1.0 × 10^−3^). The three novel associations with the strongest temporal component were cardiac dysrhythmia, pulmonary eosinophilia, and volume depletion disorder. *Conclusion*. We identified novel associations with Charcot foot in the context of pathogenesis models that include neurotrophic, neurovascular, and microtraumatic factors mediated through inflammatory cytokines. Future work should focus on confirmatory analyses. These novel areas of investigation could lead to prevention or earlier diagnosis.

## 1. Introduction

Charcot foot is a rare disease that often results in significant complications including the progressive destruction of bones and joints, loss of mobility, infections, amputations, and even death. There are approximately 40,000 new cases of Charcot foot each year. While no population-based studies of patients with Charcot foot have been described, single center prevalence estimates range from 0.1% to 0.9% [1–3].

Charcot foot most often occurs in the setting of diabetes, and an increased incidence of Charcot foot has been associated with the following: obesity, neuropathy, increased age, diabetes > 6 years, elevated hemoglobin A1c, renal failure, iron deficiency anemia, osteoporosis, and rheumatoid arthritis [4, 5]. Further, Matricali and colleagues noted a high rate of Charcot foot in simultaneous pancreas-kidney transplant patients [6], a treatment that is often used in patients with diabetes and end-stage renal failure.

Despite the well-known association of Charcot foot with diabetes and related disorders, the pathogenesis is still unknown. There are multiple theories describing the pathogenesis of Charcot foot, although the inciting mechanism is generally thought to be caused by trauma in a susceptible person with sensorimotor and/or autonomic neuropathy leading to inflammation, bone destruction, subluxation, and disordered repair in later stages.

In order to develop a better understanding of Charcot foot, we sought to use a novel approach to more extensively explore all possible diagnoses that may be associated with Charcot foot.

The increasing adoption of electronic health records (EHRs) presents the opportunity to mine vast amounts of data generated by many physicians over time in order to uncover novel associations. New computational approaches, often collectively referred to as “data mining” [7–9], have been developed that can explore large populations of patients to detect patterns or relationships that might otherwise go undetected. This may be especially true when diseases are rare and the patients are seen by a wide variety of providers and specialists, all of whom may treat aspects of the disease but may not observe some of the larger-scale patterns that might exist. Patients with Charcot foot, for example, may be seen by multiple providers including primary care physicians, podiatrists, endocrinologists, neurologists, orthopedic surgeons, and others.

Here we describe an exploratory analysis using a data mining approach applied to patients from a large, integrated health system [10, 11]. The focus of this study is not on the data mining methodology itself, but rather on the results obtained using data mining for patients who had a diagnosis of Charcot foot. We present findings that include well-known and well-established associations, which helps to confirm the validity of the approach, as well as other poorly known or even unknown associations. The technique is also able to determine the temporal relationships between Charcot foot and its associated disorders, and these relationships could have implications for explaining disease mechanisms and progression. Additionally, some of the potentially novel associations could be used to help form hypotheses about the etiology and pathogenesis of Charcot foot that could lead to future investigations.

## 2. Methods

### 2.1. Clinical Setting

This study was conducted at the University of Michigan Health System (UMHS), a large, integrated, academic, tertiary care facility with three hospitals and nearly 1,000 inpatient beds. At UMHS there are nearly 50,000 surgeries and over 45,000 inpatient visits annually. Additionally, there are almost 2 million outpatient visits per year across more than 120 outpatient clinical locations scattered primarily throughout Southeastern Michigan. There are six specialty centers including a Comprehensive Diabetes Center which houses a high volume podiatry clinic with three board certified podiatrists who specialize in the management of diabetic foot complications and amputation prevention. This study was considered exempt by our medical school's institutional review board because the dataset contained no identifiers.

### 2.2. Analytic Approach

We conducted an association analysis using international classification of disease version 9 (ICD-9) diagnosis codes among every patient in our health system. These codes had been assigned for administrative (i.e., billing/reimbursement) purposes over an approximately twenty-year period. A detailed description of the methodological approach, as well as its limitations, has been described elsewhere [10, 11]. Briefly, for each pair of ICD-9 codes (i.e., diagnoses) we calculated the chi-square statistic and its associated *P* value to determine if the two codes were significantly associated with one another. Further, we used the binomial test to determine if there was a significant temporal relationship between each pair of codes. That is, we tested if one code preceded the other code in a nonrandom manner. Such a temporal relationship can suggest, but in no way establishes, potential causality. To determine the potential role diabetes had in the associations, we also compared the frequency of diagnoses associated with Charcot foot in patients with diabetes (defined as codes 250.x, where x is one of many subcodes for diabetes) as well as those without diabetes. This was done using the proportion test. All statistical analyses were conducted using R version 2.15.3. Data were visualized with network diagrams using Cytoscape version 2.6.3 [12].

In this analysis, *P* values and statistical significance should be interpreted with several caveats in mind. The first is that no adjustments have been made for multiple hypothesis testing, which is often used in genomic analyses. With such an exploratory analysis, *P* values can be interpreted as a “ranking” in which results can be compared, and with lower *P* values suggesting more significant relationships, but we do not define any specific threshold for which a result is truly statistically significant. *P* values at or near the “traditional” threshold of 0.05 should be interpreted with caution.

### 2.3. Expert Review

The complete dataset included approximately 1.6 million patients and 41.2 million time-stamped ICD-9 codes. For the current analysis, we focused only on the patients who had been assigned the ICD-9 code 713.5 (“arthropathy associated with a neurological disorder”) that has been used in other peer-reviewed publications to describe Charcot foot [5, 13–16]. ICD-9 codes are most often used for administrative/billing purposes rather than directly for clinical care and thus can sometimes be inaccurately assigned [17]. It is not known how often the ICD-9 code 713.5 is inappropriately applied, but one study that included a small number of patients with the code 713.5 found that 92% of the cases did indeed have Charcot foot as verified by chart abstraction [16].

These associations were then independently reviewed by three Charcot foot domain experts. The associations were divided into three categories by the reviewers: (1) associations that are generally well-known, (2) associations that were potentially novel or poorly known, and (3) associations that provided minimal insight about the relationship because the codes either were nonspecific or likely represented a Charcot foot diagnosis that was initially misdiagnosed as a swollen foot. [18]. Examples of nonspecific codes were 079.99 (“viral infection, not otherwise specified”) and 693.8 (“nonspecific abnormal radiological finding of the breast”). Examples of codes that were likely misdiagnoses that were later found to be Charcot foot included 274.9 (“gout, not otherwise specified”) and 715.90 (“osteoarthrosis, unspecified site”). The decision to exclude probable misdiagnoses was supported by our observation that the misdiagnoses tended to precede the diagnosis of Charcot foot in most patients. For example, in the case of the two diagnoses described above, gout (274.9) preceded the diagnoses of Charcot foot 75% of the time and osteoarthritis (715.90) preceded Charcot foot 70% of the time. These temporal findings can be found in the Supplementary Table in the Supplementary Material (available online at http://dx.doi.org/10.1155/2014/214353).

## 3. Results

Among the overall population of 1.6 million patients, approximately 61,400 had an ICD-9 code for diabetes mellitus (ICD-9 250.x). Additionally, there were 388 patients with an ICD-9 code for Charcot foot (ICD-9 713.5), resulting in a low incidence (0.6%) even among our diabetic population. Of these 388 Charcot foot patients, 282 (72.7%) also had one of the 250.x diabetes codes assigned to them. Among the 106 patients with Charcot foot who did not have a diabetes code, there was no prevailing comorbid condition shared by the patients. The top five diagnoses appearing across the 106 patients lacking a diabetes code were ICD-9 codes 729.5 (*n* = 70; pain in limb), 719.47 (*n* = 60; pain in joint, ankle/foot), 733.90 (*n* = 58; disorder, bone/cartilage), 724.2 (*n* = 34; lumbago), and 719.46 (*n* = 33; pain in joint, lower leg).

At a large, tertiary academic medical center such as ours, it is possible that patients with rare diseases are often referred for specialized care but receive a majority of their care elsewhere. As such, it is possible that patients with Charcot foot were referred to UMHS with that diagnosis being their initial diagnosis in many cases. However, we did not find that to be the case. Twenty (5.2%) of the patients in our cohort were given a diagnosis of Charcot foot on their first visit to our health system, while an additional 10 patients (30 overall; 7.7% of cohort) were given the diagnosis within the first 30 days of visiting our health system. However, there was a wide range between the time of first contact with our health system and a diagnoses of Charcot foot: the mean time interval was 6.6 years (95% CI 6.1–7.1 years), and the median time interval was 6.2 years. It is also possible that our patients were only given a diagnosis of Charcot foot and then sent back to their referring physician outside of our network. This could cause an underrepresentation of diagnoses for those patients in our database. However, only one patient among the 388 had a single diagnosis of Charcot foot and no other diagnoses. The mean number of distinct diagnoses based on ICD-9 codes for the patients in our cohort was 84.8 (95% CI 77.9–91.8), with a median of 68.5 distinct diagnoses per patient.

**Figure 1:**
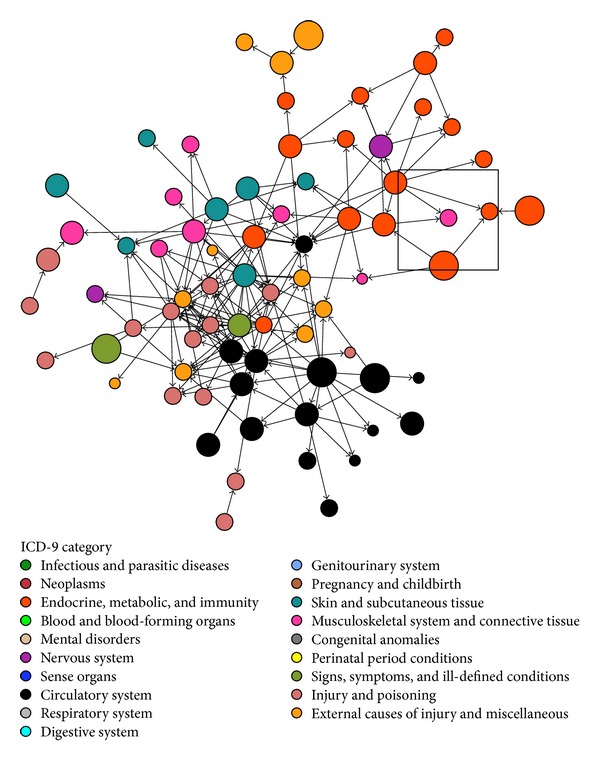
Association diagram showing the clinical milieu in which Charcot foot (pink node in center of square) often exists. Each node represents an ICD-9 code, with the size of the node proportional to its frequency in the overall dataset and node colors representing high-level clinical categories (see legend). Edges between nodes represent highly significant associations. Arrowheads show temporality with preceding nodes pointing to subsequent nodes. This figure was made using the following criteria: association *P* value < 1.0 × 10^−176^; association odds ratio > 200; temporal *P* value < 1.0 × 10^−6^. The two red nodes directly pointing to Charcot foot are related to type 2 diabetes (ICD-9 codes 250.60 and 250.90).

Our data mining approach revealed 710 pairs of associations between Charcot foot and other disorders, 676 (95.2%) of which had a *P* value for the association less than 1.0 × 10^−5^ and 603 (84.9%) of which had an odds ratio > 5.0. There were 111 (15.6%) pairs that had a significant temporal relationship with a *P* value of less than 1.0 × 10^−3^. A full list of all 710 associations with Charcot foot can be found in the Supplementary Table.  Figure 1 provides a high-level overview of some of the diagnoses associated with Charcot foot or associated with other closely related diagnoses. This figure also shows the strongest temporal relationships, using arrows between two diagnoses to represent the temporality.

A subset of known associations that were substantiated by our method is summarized in Table 1. Among these known associations, type 2 diabetes mellitus (T2DM; ICD-9 250.00) had the strongest temporal component with 87.2% of the diagnoses preceding Charcot foot when the two diagnoses cooccurred in the same patient (temporal *P* = 8.5 × 10^−37^). Many of the diagnoses presented in Table 1 are interrelated, primarily because diabetes is known to cooccur with numerous other problems including obesity, chronic kidney disease, and anemia. Further, some of the results presented in Table 1 may be misleading because they show only the frequency of single codes in the population. Thus, coding variability is not well captured in the tables. For example, code 356.9 (peripheral neuropathy) appeared in 41.5% of the patients with Charcot foot. However, when including additional codes that could represent a peripheral neuropathy (i.e., 250.60, 250.61, 250.62, 250.63, 355.8, 355.9, 356.0, 356.1, 356.9, 337.1, and 354.2), we found that these codes collectively covered 298 patients, or 76.8% of the Charcot foot population.

New or less well-known associations that were determined by expert review to be interesting in the context of current Charcot foot etiological theories, and that had a strong temporal component, are summarized in Table 2. The top three most significant associations with Charcot foot that have not been well described are related to circulatory system disorders (ICD-9 codes 414.00, 440.21, and 443.9). The top three novel/poorly known associations with the strongest temporal component were cardiac dysrhythmia (ICD-9 429.9), pulmonary eosinophilia (ICD-9 518.3), and volume depletion disorder (ICD-9 276.5). Two disorders related to lumbar disc disease were also associated with Charcot foot: (1) displacement of lumbar intervertebral disc without myelopathy (ICD-9 722.10; association *P* value 3.48 × 10^−14^; odds ratio 5.0) and (2) degeneration of lumbar or lumbosacral intervertebral disc (ICD-9 722.52; association *P* value 3.32 × 10^−54^; odds ratio 10.2). These two lumbar disc diagnoses generally preceded Charcot foot, but their levels of temporal significance were lower than our threshold for inclusion in Table 2 (temporal *P* values 0.04 and 0.01, resp.).

**Figure 2:**
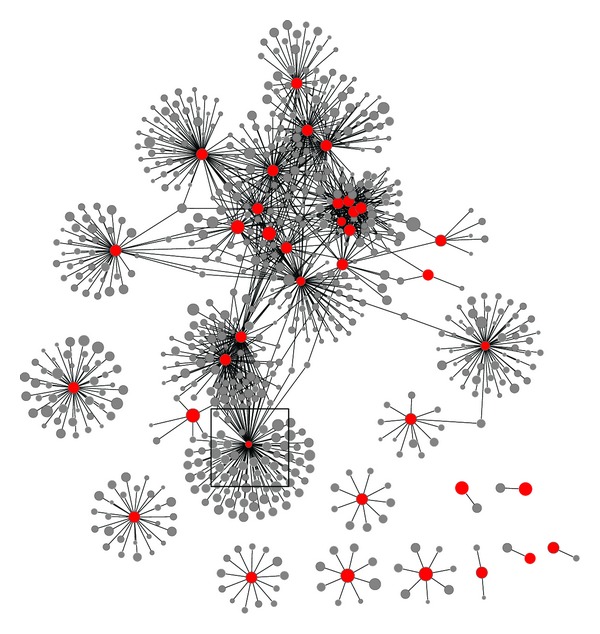
Association diagram displaying the new or poorly known diagnoses association with Charcot foot listed in Table 2 (red nodes). The red node in the center of the square is Charcot foot. Note that all of the red nodes are associated with Charcot foot, as reported in Table 2, even if they are not connected to Charcot foot in the figure. The reason is that the thresholds used to build the diagram were lower than the *P* values of some of the associations. This was done to reduce the large number of other connections that would have appeared with the nodes displayed. Temporal relationships are not shown in this figure. This figure was made using the following criteria: association *P* value < 1.0 × 10^−154^; association odds ratio > 50; temporal *P* value not considered.

Figure 2 displays an association diagram that includes the new or poorly known associations with Charcot foot that are listed in Table 2. The specific diagnoses from the table are shown as red nodes. Some of the diagnoses associated with the red nodes are highly interrelated, which can be seen by the various “clusters” and the large number of edges (lines) connecting each of the related nodes to one another. Other red nodes are less connected. These clusters suggest that there are potentially multiple etiological pathways that could lead to Charcot foot.

To demonstrate the role diabetes may have had in the relationship between Charcot foot and the diagnoses reported in Tables 1 and 2, we also report on the frequency of these diagnoses among the population of Charcot foot patients where diabetes was either present (i.e., ICD codes 250.x) or absent.  Table 3 shows the well-known associations from Table 1 stratified by diabetes, and Table 4 shows novel or poorly known associations from Table 2, also stratified by diabetes. Many of the associations (e.g., peripheral vascular disease) occurred more frequently in the diabetes cohort, whereas others were more evenly distributed among those with and without diabetes (e.g., asthma).

There were 111 association pairs that had a temporal *P* value of less than 1.0 × 10^−3^, and nearly all of them were ones in which the other disorder significantly preceded Charcot foot, using our measure of temporality which was based on the binomial test. Only four diagnoses followed Charcot foot based on the binomial test for temporal statistical significance and these were (1) obstructive sleep apnea; ICD-9 327.23; association *P* value 2.9 × 10^−24^; odds ratio 11.7; temporal *P* value 4.9 × 10^−4^, (2) abnormal chest sounds; ICD-9 786.7; association *P* value 3.2 × 10^−19^; odds ratio 10.3; temporal *P* value 9.8 × 10^−4^, (3) phantom limb syndrome; ICD-9 353.6; association *P* value < 5 × 10^−324^; odds ratio 137.5; temporal *P* value 6.6 × 10^−5^, and (4) nonspecific symptoms involving the chest and respiratory system; ICD-9 786.9; association *P* value 7.5 × 10^−132^; odds ratio 17.7; temporal *P* value 9.0 × 10^−6^.

## 4. Discussion

Data mining has been increasingly applied to clinical data [19] and has revealed multiple associations that were previously unknown [20–22]. For Charcot foot, there has been a paucity of literature that has used large databases to explore and control for comorbid conditions [5]. It is not surprising, then, that data mining techniques applied to a rare disorder such as Charcot foot might reveal new or poorly described associations. Indeed, a strength of our approach is the ability to identify associations that are not initially intuitive, without any preconceived bias. This is particularly important for Charcot foot where there is considerable debate over its etiology and pathogenesis [23, 24]. In fact, a recent 2011 international task force that was convened to summarize the Charcot foot evidence revealed few Charcot foot published studies describing its etiology and pathogenesis [25].

To better understand how Charcot foot might develop, it is helpful to discuss the findings from our study in the context of an etiological model proposed by Koeck et al. [24]. The mechanisms proposed in this model include (1) neurotrophic, (2) microtraumatic, and (3) neurovascular effects. The relationships between the model and the findings from our current study are speculative but could help shed light on a pathologic process that remains poorly understood.

Neurotrophic influences can involve local sensory loss and selective sympathetic denervation [24]. In line with this aspect of the model, we found lumbar disc disease to be associated with Charcot foot. Recent work in degenerative disc disease has implicated the role of neurotrophic factors [26]. We also found hypothyroidism to be associated with Charcot foot. Thyroid hormone deficiencies associated with brain-derived neurotrophic factor have been established [27]. Another association with Charcot foot was bladder disorder. Disruptions in afferent regulation of the bladder, as well as with brain-derived neurotrophic factor, have been implicated in bladder dysfunction [28, 29]. Overall, we found that 12 of 13 genitourinary conditions preceded the diagnosis of Charcot foot. As far as we are aware, each of the above associations is novel for Charcot foot.

Regardless of what model predominates, what is known about Charcot foot etiology is that it involves a stage of proinflammatory cytokine activity including elevated tissue necrosis factor (TNF) [30] and receptor activator nuclear factor K ligand (RANKL) [31]. This proinflammatory cytokine activity has also been described as a part of the etiology model [24, 31]. In our study, we found that coronary artery disease (CAD) demonstrated an odds ratio of 18.6 with Charcot foot. It is well established that CAD is a proinflammatory state [32]. Relatedly, we also found hyperlipidemia to be associated with Charcot foot. Treatment of CAD with 3-hydroxy-3-methylglutaryl-coenzyme A (HMGCoA) reductase inhibitors (i.e., “statins”) has also been described to have an anti-inflammatory effect [33, 34]. CAD has not been described in prior Charcot foot multivariate models [4, 5]. It could be that age and diabetes status are collinear with CAD using a multivariate approach.

Other associations that do not fit neatly into any model are also worth mentioning including the association between Charcot foot and alkalosis, which had an odds ratio of 11.2. Transient perturbations in parathyroid hormone, calcium, and phosphorus [35, 36] may explain why alkalosis preceded Charcot foot 100% of the time. We also found pulmonary eosinophilia to be associated with Charcot foot, and this relationship had the fourth smallest *P* value for all the temporal associations we described. Treatment for pulmonary eosinophilia with inhaled [37] or oral steroids [38] and subsequent bone mineral density and bone metabolism changes could partly explain why this disorder was associated with Charcot foot. Furthermore, we found an association with esophageal reflux and Charcot foot. This association could be due to concurrent use of proton pump inhibitors that have been described to reduce bone mineral density [39]. As far as the authors are aware, the associations described above are also novel in Charcot foot.

While we describe several new associations with Charcot foot, our analysis also confirmed other known risk factors for Charcot foot. For example, we found associations of Charcot foot with obesity, peripheral neuropathy, decreased bone mineral density, and a history of pancreas and/or kidney transplant surgery [4]. Our findings also agree with Stuck and colleagues in finding associations with renal failure, rheumatoid arthritis, and anemia [5].

Our approach did have limitations. One of the main limitations is that we chose to discuss those new associations that fit our conceptual model. Our discussion was not exhaustive of the 111 significant temporal associations we described using our conservative acceptance of *P* value thresholds. Thus, all of the associations are provided as a Supplementary Table. The data presented here can be used for hypothesis generation, not to make any firm conclusions about causality. The more interesting associations could potentially be followed up by a chart review of the clinical records to ascertain more details about the relationships, as has been done with other interesting associations [20].

It is also important to note that our analysis focused on ICD-9 codes, which have potential limitations in not only accuracy but also specificity. Some codes are nonspecific and therefore do not provide enough detail to fully understand what they represent. For example, in our population the code 733.90 (bone/cartilage disorder, not otherwise specified) was present in 60.1% of the patients with Charcot foot. From the code itself we are unable to determine if these represent a different condition from Charcot foot. Using the temporal data to provide additional insight, we can observe that in the majority of patients the bone/cartilage disorder preceded the diagnoses of Charcot foot, suggesting that 733.90 could have been an initial misdiagnosis of Charcot foot rather than a distinct clinical entity. We also found that in some cases groups of codes should be considered collectively rather than separately. This is the case for the diabetes codes as well as those potentially representing peripheral neuropathy. Additionally, some of the variation in the use of codes could represent differences in coding among disciplines, since the codes used in our analysis were assigned not just by podiatrists but by clinicians across the entire health system.

Another limitation of our study is that medications were not included in our analysis, and yet these could influence both the development and prevention of Charcot foot. Future work should include additional clinical features to develop a richer understanding of Charcot foot and its etiology. Further, it is possible that some patients with true Charcot foot continued to remain misdiagnosed (with gout or osteoarthropathy, e.g.) and thus never received the diagnosis code of 713.5. Future studies should examine such cases for potential misdiagnoses of Charcot foot, especially because earlier detection could provide substantial clinical benefit [40].

A primary purpose for presenting our results was to help generate hypotheses for further testing and confirmation and to better shed light on Charcot foot, for which the etiology remains poorly described. Future work should seek to confirm the validity of the associations found in our exploratory analysis. One next step would be to conduct a detailed chart review of the cases from the electronic health record to better understand the context in which the associations occurred and even to confirm the diagnosis of Charcot foot. Incorporating other data sources, such as from the Veterans Health Administration or the Centers for Medicare and Medicaid Services, could also provide additional opportunities for analysis of this rare disorder. It will also be important to understand in more detail how coding practices (and variability) could influence our findings.

## 5. Conclusion

We present here a novel application of an approach to mining large databases, such as those available through the electronic health record, for elucidating new associations with Charcot foot that might otherwise go unnoticed. We also uniquely describe the temporal relationships between these various diseases and Charcot foot. This approach has potential for generating new hypotheses regarding the cause of Charcot foot. At a minimum, we hope to create a heightened awareness of Charcot foot in patients with associated conditions to help identify these at-risk patients sooner. Also, if we can elucidate the pathway of Charcot foot, it would hopefully lead to better treatment modalities including drug development.

## Supplementary Material

The Supplementary Material contains a table in the Microsoft Excel format that lists all of the associations we found with Charcot foot. These include associations that were found but not discussed in the manuscript. Additional information about how to interpret the results in the supplementary table are also provided within the file.

## Figures and Tables

**Table 1:** Well-known associations with Charcot foot, based on 388 patients with Charcot foot. Although not all associations presented here show a significant temporal relationship with Charcot foot, those that do preceded the diagnosis of Charcot foot.

Disease category	ICD-9 code	ICD-9 description	Number (%) with both disorders	Association odds ratio	Association *P* value	Temporal *P* value
Endocrine, nutritional and metabolic diseases, and immunity disorders	250.00*	Type 2 diabetes mellitus	243 (62.6)	55.4	<5 × 10^−324^	8.5 × 10^−37^
	278.00	Obesity	52 (13.4)	11.7	2.4 × 10^−95^	4.1 × 10^−4^

Nervous system	356.9	Peripheral neuropathy	161 (41.5)	101.2	<5 × 10^−324^	3.9 × 10^−12^

Blood and blood-forming organs	280.0	Anemia due to chronic blood loss	16 (4.1)	9.9	1.0 × 10^−26^	0.80
	285.9	Anemia, not otherwise specified	98 (25.3)	14.9	3.3 × 10^−205^	0.085
	285.29	Anemia of other chronic diseases	10 (2.6)	43.1	1.1 × 10^−79^	1
	280.9	Iron deficiency anemia	32 (8.2)	14.5	1.5 × 10^−79^	0.60

Skin and subcutaneous tissue	682.6	Cellulitis/abscess of leg	87 (22.4)	41.9	<5 × 10^−324^	4.4 × 10^−6^
	682.7	Cellulitis/abscess of foot	98 (25.3)	133.8	<5 × 10^−324^	5.5 × 10^−6^

Genitourinary system	593.9	Renal & ureteral disorder, not otherwise specified	119 (30.7)	20.9	<5 × 10^−324^	4.5 × 10^−4^
	585	Chronic kidney disease	64 (16.5)	29.4	7.7 × 10^−314^	2.4 × 10^−5^

Musculoskeletal system and connective tissue	714.0	Rheumatoid arthritis	37 (9.5)	13.5	1.2 × 10^−83^	1.3 × 10^−5^
	733.00	Osteoporosis	44 (11.3)	12.1	4.4 × 10^−86^	0.29
	733.90	Bone/cartilage disorder, not otherwise specified	233 (60.1)	26.0	<5 × 10^−324^	5.9 × 10^−12^

Injury and poisoning	996.81	Complication of kidney transplant	44 (11.3)	43.3	<5 × 10^−324^	3.9 × 10^−4^

External causes of injury and supplemental classification	V42.0	Kidney transplant	50 (12.9)	42.1	<5 × 10^−324^	9.0 × 10^−5^

*This table only lists ICD-9 code 250.00, not the other diabetes codes that also appeared in this cohort of patients.

**Table 2:** Novel or poorly known associations with Charcot foot based on 388 patients with Charcot foot. All disorders listed in this table temporally preceded Charcot foot in a statistically significant (*P* < 0.001) number of patients.

Disease category	ICD-9 code	ICD-9 description	Number (%) with both disorders	Association odds ratio	Association *P* value	Temporal *P* value
Endocrine, nutritional and metabolic diseases, and immunity disorders	244.9	Hypothyroidism	42 (10.8)	6.1	3.2 × 10^−35^	9.4 × 10^−4^
	272.0	Hypercholesterolemia	82 (21.1)	17.9	7.0 × 10^−223^	3.2 × 10^−5^
	272.4	Hyperlipidemia	173 (44.6)	17.4	6.4 × 10^−321^	1.1 × 10^−12^
	276.3	Alkalosis	13 (3.4)	14.0	4.1 × 10^−32^	2.4 × 10^−4^
	276.5	Volume depletion disorder	84 (21.6)	8.1	3.0 × 10^−90^	5.0 × 10^−15^

Sense organs	366.16	Senile nuclear cataract	110 (28.4)	13.3	3.2 × 10^−195^	7.7 × 10^−4^

Circulatory system	401.1	Benign essential hypertension	113 (29.1)	13.3	3.3 × 10^−197^	1.2 × 10^−5^
	402.10	Benign hypertensive disease without heart failure	15 (3.9)	6.6	1.9 × 10^−15^	9.8 × 10^−4^
	411.1	Intermediate coronary syndrome	38 (9.8)	13.9	1.2 × 10^−88^	1.2 × 10^−4^
	413.9	Angina pectoris	41 (10.6)	13.1	8.2 × 10^−89^	1.1 × 10^−4^
	414.0	Coronary atherosclerosis	18 (4.6)	7.2	2.2 × 10^−20^	7.6 × 10^−6^
	414.00	Coronary atherosclerosis, vessel type	155 (39.9)	18.6	<5 × 10^−324^	4.5 × 10^−10^
	414.01	Coronary atherosclerosis, native artery	97 (25.0)	16.3	2.2 × 10^−226^	8.1 × 10^−6^
	414.02	Coronary atherosclerosis, autologous graft	11 (2.8)	15.7	1.1 × 10^−30^	9.8 × 10^−4^
	414.9	Chronic ischemic heart disease	49 (12.6)	14.7	2.5 × 10^−118^	6.2 × 10^−7^
	424.0	Mitral valve disorder	32 (8.2)	7.4	8.4 × 10^−36^	1.1 × 10^−4^
	425.4	Cardiomyopathy, primary	44 (11.3)	11.6	2.7 × 10^−82^	2.5 × 10^−5^
	427.9	Cardiac dysrhythmia	168 (43.3)	7.6	1.5 × 10^−119^	1.7 × 10^−31^
	428.0	Congestive heart failure	84 (21.6)	11.0	1.5 × 10^−129^	2.4 × 10^−6^
	428.9	Heart failure, not otherwise specified	38 (9.8)	10.1	5.6 × 10^−61^	4.7 × 10^−4^
	429.9	Heart disease, not otherwise specified	39 (10.1)	10.5	9.3 × 10^−66^	3.4 × 10^−7^
	440.21	Atherosclerosis of native arteries of the extremities with intermittent claudication	93 (24.0)	48.5	<5 × 10^−324^	5.3 × 10^−5^
	443.9	Peripheral vascular disease	95 (24.5)	38.5	<5 × 10^−324^	1.9 × 10^−4^

Respiratory system	465.9	Acute upper respiratory infection	93 (24.0)	2.7	2.0 × 10^−17^	1.9 × 10^−11^
	466.0	Acute bronchitis	75 (19.3)	5.5	4.7 × 10^−49^	2.2 × 10^−6^
	473.9	Chronic sinusitis	35 (9.0)	4.4	4.3 × 10^−19^	1.2 × 10^−4^
	477.9	Allergic rhinitis	40 (10.3)	2.3	8.5 × 10^−07^	1.8 × 10^−4^
	493.90	Asthma (without status asthmaticus)	46 (11.9)	3.0	2.2 × 10^−13^	1.8 × 10^−6^
	518.3	Pulmonary eosinophilia	125 (32.2)	7.1	1.1 × 10^−97^	1.8 × 10^−22^

Digestive system	530.81	Esophageal reflux	64 (16.5)	4.7	2.2 × 10^−35^	7.7 × 10^−5^
	560.9	Intestinal obstruction, not otherwise specified	30 (7.7)	6.5	1.1 × 10^−28^	3.2 × 10^−4^

Genitourinary system	596.9	Bladder disorder, not otherwise specified	12 (3.1)	7.3	3.1 × 10^−14^	4.9 × 10^−4^
	607.84	Impotence, organic origin	33 (8.5)	14.0	2.1 × 10^−78^	3.2 × 10^−4^

Skin and subcutaneous tissue	692.9	Contact dermatitis	52 (13.4)	3.3	3.5 × 10^−17^	3.6 × 10^−5^

Musculoskeletal system and connective tissue	724.2	Lumbago	117 (30.2)	6.7	2.0 × 10^−86^	8.9 × 10^−11^
	729.1	Myalgia/myositis, not otherwise specified	55 (14.2)	9.1	2.9 × 10^−74^	3.6 × 10^−4^
	715.90	Osteoarthrosis, not otherwise specified	99 (25.5)	17.3	2.9 × 10^−245^	1.1 × 10^−4^

Injury and poisoning	860.0	Traumatic pneumothorax	27 (7.0)	6.7	6.2 × 10^−27^	3.1 × 10^−4^
	847.0	Sprain/strain of neck	21 (5.4)	5.7	1.5 × 10^−17^	2.2 × 10^−4^

**Table 3:** Well-known associations with Charcot foot, compared between those with (*n* = 282) and without (*n* = 106) diabetes, defined as any ICD-9 250.x code. The associations shown here are based on the ones shown in Table 1.

Disease category	ICD-9 code	ICD-9 description	Number (%) among those with diabetes	Number (%) among those without diabetes	*P* value for difference in proportions
Endocrine, nutritional and metabolic diseases, and immunity disorders	278.00	Obesity	46 (16.3)	6 (5.7)	10.0 × 10^−3^

Nervous system	356.9	Peripheral neuropathy	143 (50.7)	18 (17.0)	3.80 × 10^−9^

Blood and blood-forming organs	280.0	Anemia due to chronic blood loss	14 (5.0)	2 (1.9)	0.28
	285.9	Anemia, not otherwise specified	88 (31.2)	10 (9.4)	2.0 × 10^−5^
	285.29	Anemia of other chronic diseases	8 (2.8)	2 (1.9)	0.87
	280.9	Iron deficiency anemia	29 (10.3)	3 (2.8)	0.030

Skin and subcutaneous tissue	682.6	Cellulitis/abscess of leg	80 (28.4)	7 (6.6)	8.8 × 10^−6^
	682.7	Cellulitis/abscess of foot	94 (33.3)	4 (3.8)	5.2 × 10^−9^

Genitourinary system	593.9	Renal & ureteral disorder, not otherwise specified	111 (39.4)	8 (7.5)	3.0 × 10^−9^
	585	Chronic kidney disease	63 (22.3)	1 (0.9)	9.3 × 10^−7^

Musculoskeletal system and connective tissue	714.0	Rheumatoid arthritis	28 (9.9)	9 (8.5)	0.81
	733.00	Osteoporosis	36 (12.8)	8 (7.5)	0.21
	733.90	Bone/cartilage disorder, not otherwise specified	175 (62.1)	58 (54.7)	0.23

Injury and poisoning	996.81	Complication of kidney transplant	43 (15.2)	1 (0.9)	1.6 × 10^−4^

External causes of injury and supplemental classification	V42.0	Kidney transplant	49 (17.4)	1 (0.9)	3.6 × 10^−5^

**Table 4:** Novel or poorly know associations with Charcot foot, compared between those with (*n* = 282) and without (*n* = 106) diabetes, defined as any ICD-9 250.x code. The associations shown here are based on the ones shown in Table 2.

Disease category	ICD-9 code	ICD-9 description	Number (%) among those with diabetes	Number (%) among those without diabetes	*P* value for difference in proportions
Endocrine, nutritional and metabolic diseases, and immunity disorders	244.9	Hypothyroidism	34 (12.1)	8 (7.5)	0.28
	272.0	Hypercholesterolemia	78 (27.7)	4 (3.8)	5.9 × 10^−7^
	272.4	Hyperlipidemia	163 (57.8)	10 (9.4)	<2.2 × 10^−16^
	276.3	Alkalosis	11 (3.9)	2 (1.9)	0.51
	276.5	Volume depletion disorder	75 (26.6)	9 (8.5)	2.0 × 10^−4^

Sense organs	366.16	Senile nuclear cataract	102 (36.2)	8 (7.5)	5.10 × 10^−8^

Circulatory system	401.1	Benign essential hypertension	103 (36.5)	10 (9.4)	3.3 × 10^−7^
	402.10	Benign hypertensive disease without heart failure	14 (5.0)	1 (0.9)	0.12
	411.1	Intermediate coronary syndrome	38 (13.5)	0 (0.0)	1.5 × 10^−4^
	413.9	Angina pectoris	40 (14.2)	1 (0.9)	3.2 × 10^−4^
	414.0	Coronary atherosclerosis	18 (6.4)	0 (0.0)	0.017
	414.00	Coronary atherosclerosis, vessel type	143 (50.7)	12 (11.3)	3.9 × 10^−12^
	414.01	Coronary atherosclerosis, native artery	89 (31.6)	8 (7.5)	2.2 × 10^−6^
	414.02	Coronary atherosclerosis, autologous graft	11 (3.9)	0 (0.0)	0.086
	414.9	Chronic ischemic heart disease	49 (17.4)	0 (0.0)	9.88 × 10^−6^
	424.0	Mitral valve disorder	31 (11.0)	1 (0.9)	2.7 × 10^−3^
	425.4	Cardiomyopathy, primary	42 (14.9)	2 (1.9)	6.2 × 10^−4^
	427.9	Cardiac dysrhythmia	143 (50.7)	25 (23.6)	2.7 × 10^−6^
	428.0	Congestive heart failure	78 (27.7)	6 (5.7)	5.4 × 10^−6^
	428.9	Heart failure, not otherwise specified	36 (12.8)	2 (1.9)	2.5 × 10^−3^
	429.9	Heart disease, not otherwise specified	38 (13.5)	1 (0.9)	5.2 × 10^−4^
	440.21	Atherosclerosis of native arteries of the extremities with intermittent claudication	89 (31.6)	4 (3.8)	2.4 × 10^−8^
	443.9	Peripheral vascular disease	91 (32.3)	4 (3.8)	1.3 × 10^−8^

Respiratory system	465.9	Acute upper respiratory infection	71 (25.2)	22 (20.8)	0.44
	466.0	Acute bronchitis	60 (21.3)	15 (14.2)	0.15
	473.9	Chronic sinusitis	26 (9.2)	9 (8.5)	0.98
	477.9	Allergic rhinitis	30 (10.6)	10 (9.4)	0.87
	493.90	Asthma (without status asthmaticus)	34 (12.1)	12 (11.3)	0.98
	518.3	Pulmonary eosinophilia	104 (36.9)	21 (19.8)	2.0 × 10^−3^

Digestive system	530.81	Esophageal reflux	47 (16.7)	17 (16.0)	1
	560.9	Intestinal obstruction, not otherwise specified	25 (8.9)	5 (4.7)	0.25

Genitourinary system	596.9	Bladder disorder, not otherwise specified	9 (3.2)	3 (2.8)	1
	607.84	Impotence, organic origin	29 (10.3)	4 (3.8)	0.065

Skin and subcutaneous tissue	692.9	Contact dermatitis	38 (13.5)	14 (13.2)	1

Musculoskeletal system and connective tissue	724.2	Lumbago	83 (29.4)	34 (32.1)	0.70
	729.1	Myalgia/myositis, not otherwise specified	45 (16.0)	10 (9.4)	0.14
	715.90	Osteoarthrosis, not otherwise specified	74 (26.2)	25 (23.6)	0.69

Injury and poisoning	860.0	Traumatic pneumothorax	25 (8.9)	2 (1.9)	0.029
	847.0	Sprain/strain of neck	15 (5.3)	6 (5.7)	1
